# Lifestyle and cardiovascular risk factors in a Swedish primary care population with self-reported psychiatric symptoms

**DOI:** 10.1016/j.pmedr.2023.102547

**Published:** 2023-12-12

**Authors:** Veronica Milos Nymberg, Peter Nymberg, Miriam Pikkemaat, Susanna Calling, Emelie Stenman, Anton Grundberg, J. Gustav Smith, Kristina Sundquist

**Affiliations:** a Center for Primary Health Care Research, Department of Clinical Sciences Malmö, Lund University, Malmö, Sweden; b Primary Care Skåne, Region Skåne, Sweden; c School of Health and Welfare, Halmstad University, Sweden; d Department of Cardiology, Clinical Sciences, Lund University and Skåne University Hospital, Lund, Sweden; e Department of Molecular and Clinical Medicine, Institute of Medicine, University of Gothenburg, Gothenburg, Sweden; f Wallenberg Center for Molecular Medicine and Lund University Diabetes Center, Lund University, Lund, Sweden

**Keywords:** Lifestyle, Psychiatric symptoms, Primary care, Targeted Health Dialogues, Psychiatric illness

## Abstract

**Objective:**

Individuals with psychiatric illness suffer from poorer physical health compared with the general population and have a higher risk of developing cardiovascular and metabolic diseases. This cross-sectional study aims to describe the prevalence of lifestyle and cardiovascular risk factors and the association with self-reported psychiatric symptoms in a population of 40-year-old individuals screened with targeted Health Dialogues in southern Sweden.

**Methods:**

All 40-year-old individuals registered at 99 primary healthcare centers in southern Sweden were invited to participate. Self-reported lifestyle habits on a web questionnaire, anthropometric measurements, blood pressure, and blood tests were collected. The Health Dialogue resulted in a risk level assessment for different lifestyle habits and a meeting with a trained coach.

**Results:**

A total of 1831 individuals completed a Health Dialogue between 1st January 2021 and 30th June 2022. There were more individuals with high-risk levels for several lifestyle habits in the group with self-reported psychiatric illness compared with the rest of the study population. The analysis showed that physical inactivity, unhealthy diet, high-risk alcohol intake, tobacco use, psychosocial strain, higher BMI, and waist-hip ratio were associated with increased levels of psychiatric symptoms after adjustment for sex and socioeconomic factors.

**Conclusion:**

Unhealthy lifestyle habits were associated with self-reported psychiatric symptoms in 40-year-old individuals assessed with targeted Health Dialogues in a primary care context. Organized screening might contribute to early detection of modifiable risk factors for cardiovascular disease. Individuals with psychiatric symptoms should be prioritized for screening of unhealthy lifestyle behaviors.

## 1 Introduction

Individuals with psychiatric illness suffer from poorer physical health compared with the general population, a higher risk of developing cardiovascular and metabolic diseases (Stanley and Laugharne, 2012, Firth et al., 2019) and a higher mortality rate (Walker et al., 2015). We have convincing evidence today that not only patients with severe psychiatric illness, such as bipolar disorder or schizophrenia, but also individuals with psychiatric symptoms from the entire spectrum of mental disorders experience reduced life expectancy compared with the rest of the population (Firth et al., 2019). Anxiety and depression have been associated with incident cardiovascular disease (CVD) (Batelaan et al., 2016), hypertension (Pan et al., 2015), diabetes (Osborn et al., 2008) and cancer (Jia et al., 2017). Anxiety disorders are associated with a higher risk for cardiovascular events, including stroke, coronary heart disease, heart failure and cardiovascular death (Emdin et al., 2016). Common stress disorder has also been associated with an increased risk for CVD (Gradus et al., 2015). A Swedish study showed that several health problems, including stress symptoms and pain, were associated with an increased risk for myocardial infarction in middle-aged women (Calling et al., 2018).

Some of the increased risks for CVD in people suffering from psychiatric illness, as compared to the rest of the population, may be related to the metabolic effects of psychotropic medication (Himmerich et al., 2015). Another major contributing factor is an unhealthy lifestyle, such as sedentary behavior, poor diet, smoking, alcohol, or substance abuse (Firth et al., 2019). Therefore, early preventive interventions focused on lifestyle modification have been advocated (Firth et al., 2019, Stanley and Laugharne, 2014, Firth et al., 2020), with several key components being proposed, such as smoking cessation (Curtis et al., 2018), increased physical activity (Stubbs et al., 2016), or improved diet quality (Teasdale et al., 2018, Burrows et al., 2022).

Several lifestyle interventions have been performed in people with severe psychiatric illness (Naslund et al., 2017, Deenik et al., 2019). Meanwhile, a large majority of psychiatric consultations for depression, anxiety, stress, and sleep disorders occur in primary care (Sundquist et al., 2017). Although a growing body of evidence has linked symptoms of different psychiatric disorders to poor lifestyle habits such as physical inactivity, tobacco smoking, and unhealthy dietary habits (Firth et al., 2020), there are knowledge gaps about these links in preventive lifestyle interventions among individuals with psychiatric illness in primary health care settings (Firth et al., 2020). Individualized lifestyle programs seem to be more effective on modifiable health and cardiovascular risk factors compared with lifestyle screening in the general population (Lingfors et al., 2009, Ronngren et al., 2018). To individualize lifestyle counselling in primary care, targeted Health Dialogues have been developed and introduced in several Swedish regions during recent years (Lingfors et al., 2003, Norberg et al., 2010). The method has been previously shown to reduce cardiovascular mortality (Blomstedt et al., 2015) as well as all-cause mortality (Blomstedt et al., 2015, Lingfors and Persson, 2019) in large prospective cohort studies and has been proven cost-effective (Lindholm et al., 2018). In southern Sweden, a large screening cohort study started in 2021, in which 40- and 50-year-old individuals are invited to a targeted Health Dialogue at their primary health care center (PHCC).

This cross-sectional study aims to describe the prevalence of unhealthy lifestyle habits and cardiovascular risk factors and the association with self-reported psychiatric symptoms in 40-year-old individuals screened with targeted Health Dialogues.

## 2 Methods

### 2.1 Patients and settings

Region Scania is located in southern Sweden, and includes both rural and urban areas, with a total of 1.4 million inhabitants. The primary healthcare service in the region is supplied by 170 tax-funded private or public PHCCs. In 2021, a total of 99 PHCCs had implemented targeted Health Dialogues. All 40-year-old individuals, which were listed at these 99 PHCCs, were invited on 1st January 2021 to participate in the project. This cross-sectional study is based on data collected from the 40-year-old individuals between 1st January 2021 and 30th June 2022 who provided written consent to participate in the study and completed a Health Dialogue.

### 2.2 The Targeted Health Dialogue

**Fig. 1:**
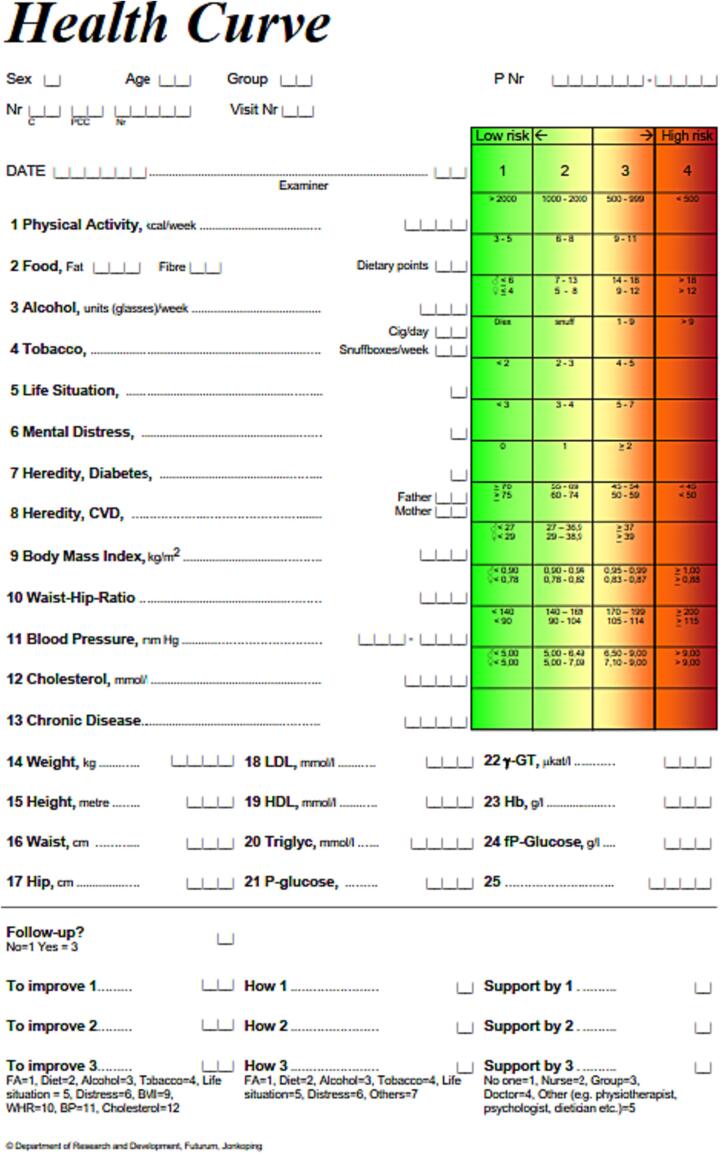
The Health Profile (in English).

The structured method for targeted Health Dialogue has previously been validated in a Swedish primary care context to detect unhealthy lifestyle habits (Lingfors et al., 2003). Health dialogues in the current study were all conducted by Health Dialogue coaches trained in motivational interviewing, with medical professionals such as registered nurses, physicians, dieticians, occupational therapists or physiotherapists participating. 40-year-old individuals were invited to a Health Dialogue with a letter containing information about the screening and an informed consent form about participating in a prospective cohort study. Prior to the Health Dialogue, blood samples were collected from peripheral venipuncture and serum samples underwent analysis using routine clinical chemistry laboratory instruments for glucose, cholesterol, high-density-lipoprotein cholesterol (HDL-C), and low-density-lipoprotein-cholesterol (LDL-C). Patients filled in a web-based detailed questionnaire with questions regarding different lifestyle habits (diet, physical activity, tobacco use, alcohol intake, mental stress, and psychosocial strain) as well as heredity for CVD or diabetes. During the Health Dialogue at the PHCC, BMI, waist-hip ratio and blood pressure were measured. The results of blood tests, questionnaires and measurements were aggregated in a visual, colorful scale showing a risk assessment for every lifestyle habit, with risk levels between 1 and 4 or 1–3, with three or four being the highest risk level on the Health Profile (Fig. 1). The Health Profile functions as a prognostic tool, estimating the cardiovascular risk with the current lifestyle and facilitating a discussion during the Health Dialogue between the patient and the Health Dialogue coach about possible actions to be taken (such as smoking cessation, alcohol intake, counselling, or referral to another health care professional). Referrals to other specialists at the PHCC or to other clinics were sent when needed.

### 2.3 Measurements

#### 2.3.1 Psychiatric symptoms

The level of psychiatric illness is assessed on the Health Profile using a score calculated by adding points from the following questions: 1) During the last 12 months, have you experienced some of the following symptoms: sleep problems, anxiety, depression, or general fatigue? (Yes/No, the answer Yes gives one point for every symptom), and 2) Mark on this line which level of stress you have experienced during the past year (0–19 mm gives 0 points, 20–34 mm gives one point, 35–44 gives two points, 45–50 mm gives three points). The total score is the sum of all points, and results in three levels of psychiatric illness on the Health Profile: level 1 (green, <3 points, low risk), level 2 (yellow, 3–4 points, medium risk) and level 3 (red, 5–7 points, high risk). On the Health Profile, this health behavior is called Mental Distress (Fig. 1). We categorized the patients into two groups: one group including levels 1 and 2, and one group including the highest level of psychiatric illness, level 3.

#### 2.3.2 Unhealthy lifestyle habits

The cut off-lines used in the assessment of risk levels for different lifestyle habits were those recommended by the Swedish National Board of Health and Welfare in 2018 (Nationella, 2018) and used in previously published studies with targeted Health Dialogues (Lingfors and Persson, 2019).

Physical activity was assessed with questions about exercise conducted during leisure time with four response categories (sedentary leisure time, moderate exercise, strenuous exercise, hard exercise), the mode of transportation to work and the amount of activity minutes during the last week. The cut off-line for insufficient physical activity was drawn at less than 2000 kcal per week in leisure time.

Dietary habits were assessed with questions about food quality and different food types, using a fat- and fiber index. Participants who reported intake of ‘sweets, chocolate, or sugar-sweetened drinks’ ≥ 2 times per day or ‘cakes or cookies’ ≥ 2 times per day, or both categories once daily, were considered as having poor dietary habits regardless of the fat- and fiber index.

High-risk alcohol habits were defined as intake of ≥ 5 (women) or ≥ 7 (men) standard glasses of alcohol (equivalent to 12–15 cl wine) per week. Drinking ≥ 4 (women) or ≥ 5 (men) standard glasses per occasion at least monthly was also categorized as a high-risk alcohol intake behavior.

Tobacco use was defined as smoking cigarettes or using ‘snus’ (Swedish oral tobacco product), e-cigarettes or waterpipe regularly.

Life situation was assessed using questions about marital status, social network, employment, risk of unemployment within a year or concerns/worries about personal economy.

Socioeconomic factors considered to be relevant for the analysis were: marital status (married or cohabiting, yes/no), employment (employed, self-employed, retired, unemployed, other) and educational level (≤9 years, 9–12 years, >12 years).

#### 2.3.3 Diagnoses

For further sub-analysis of the material, diagnosis codes were collected from Region Scania’s Health Care Utilization Database Scania (RSVD). Diagnosis codes were classified according to the International Classification of Diseases ICD-10 for psychiatric diseases (F), hypertension (I10 and I11) and diabetes mellitus type 2 (E11).

#### 2.3.4 Ethical approvall

The study met the institution's guidelines for protection of human subjects concerning safety and privacy. The corresponding author warrants that ethical approval has been obtained from the Swedish Ethical Review Authority (registration number 2020-02689 with later amendments). The study was registered at ClinicalTrials.gov, identifier: NCT04912739.

### 2.4 Statistical analysis

The prevalence of cardiovascular and metabolic risk in participants with and without high levels of psychiatric symptoms on the Health Profile is presented with descriptive measures (mean, percentage). Group comparisons were made with Pearson’s χ ^2^ tests for categorical variables and Student’s *t*-test or Mann-Whitney test for continuous variables. The association between different lifestyle habits on the Health Profile (independent variable) and level of psychiatric symptoms (dependent variable) was studied with logistic regression analysis. The results are shown as odds ratios (ORs), using a confidence interval (CI) of 95 %, and a significance level of 0.05. The association was adjusted for sex, marital status, employment, and educational level. All statistical analyses were performed with R version 4.2.1 (R Core Team, 2022).

## 3 Results

**Fig. 2:**
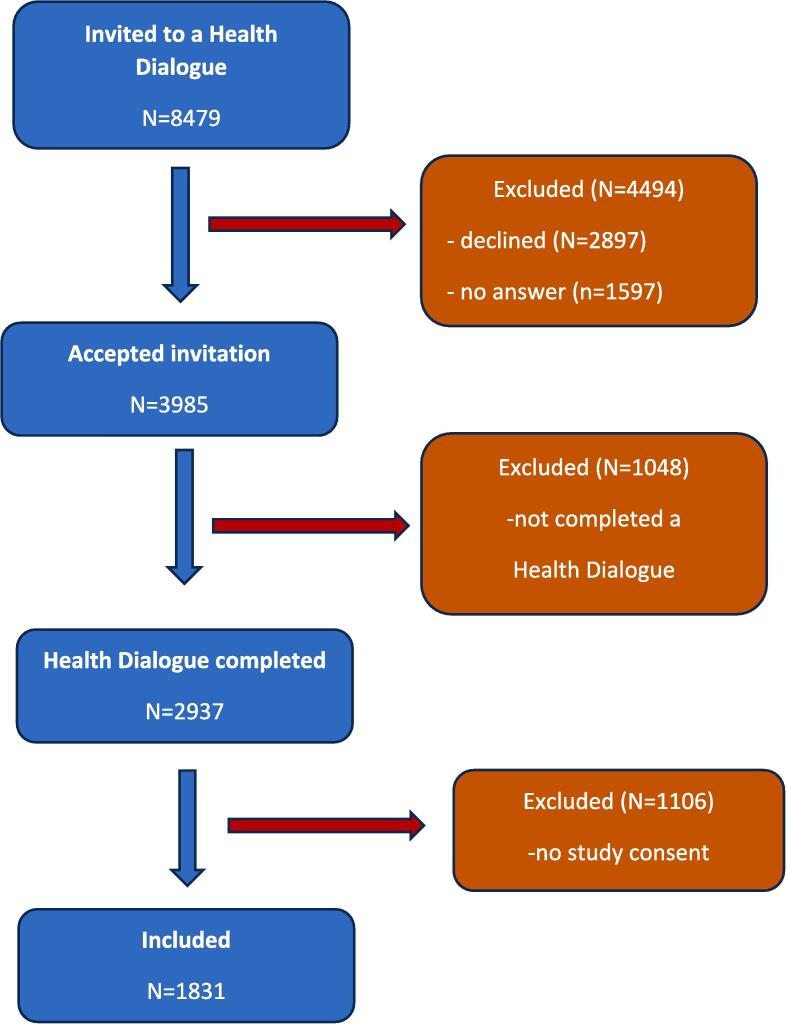
The flow chart for the recruitment of the study cohort, in which 40-year-old individuals were invited to a targeted Health Dialogue.

A total of 8479 individuals from 99 PHCCs were invited to participate in a targeted Health Dialogue, of which 3985 (47 %) accepted to participate in the study. Of these, 1831 (46 %) completed a Health Dialogue and provided written informed consent. They represented 21 % of the invited individuals. The inclusion flow is presented in Fig. 2.

619 individuals (34 %) reported level 1 for psychiatric symptoms, 578 (32 %) reported level 2 and 634 reported (35 %) level 3, which is the highest risk level for psychiatric symptoms on the Health Profile. Sex distribution, assessment of lifestyle habits and metabolic risk factors are presented in Table 1. A total of 83 (4.5 %) participants had a previously registered hypertension diagnosis (I.10 and I.11 according to ICD-10) and 29 (1.5 %) had diabetes mellitus type 2 (E.11). There was no difference between the groups with or without psychiatric symptoms regarding the prevalence of hypertension or diabetes.

**Table 1:** Comparisons between the groups for psychiatric symptoms regarding metabolic risk factors and risk levels for different lifestyle habits.

Variable	Total N = 1831(%)	Level of self-reported psychiatric symtoms on the Health Profile		p-value
		1–2	3	
Sex				<0.001
Men, N (%)	821 (45)	589 (49)	232 (37)	
Women, N (%)	1010 (55)	608 (51)	402 (63)	
Systolic blood pressure, mean (SD)	122 (13)	122 (13)	122 (13)	0.65
Diastolic blood pressure, mean (SD)	79 (10)	78 (10)	79 (10)	0.20
Blood pressure – risk level, N (%)				0.93
1 (Green) (<140/90 mmHg)	1488 (81)	970 (81)	518 (82)	
2 (140–169/90–104 mmHg)	314 (17)	208 (17)	106 (17)	
3 (≥170-199/105–114 mmHg)	17 (0.9)	11 (0.9)	6 (0.9)	
4 (Red) (≥200/115 mmHg)	8 (0.4)	6 (0.5)	2 (0.3)	
Missing	4 (0.2)	2 (0.2)	2 (0.3)	
S-LDL, mean (SD)	3.29 (0.95)	3.28 (0.92)	3.32 (0.99)	0.41
S-HDL, mean (SD)	1.39 (0.41)	1.39 (0.40)	1.40 (0.42)	0.64
Total cholesterol, mean (SD)	4.78 (0.88)	4.76 (0.85)	4.82 (0.93)	0.22
Cholesterol – risk level, N (%)				0.55
1 (Green)	1108 (61)	727 (61)	381 (60)	
2	652 (36)	428 (36)	224 (35)	
3	58 (3.2)	33 (2.8)	25 (4.0)	
4 (Red)	2 (0.1)	1 (0.1)	1 (0.2)	
Missing	11 (0.6)	8 (0.7)	3 (0.5)	
F-plasma glucose, mean (SD)	5.44 (0.79)	5.44 (0.81)	5.43 (0.74)	0.94
F-plasma glucose, N (%)				0.16
≤ 6 mmol/l	1647 (90)	1067 (89)	580 (91)	
6.1–6.9 mmol/l	133 (7.3)	97 (8.2)	36 (5.7)	
≥ 7 mmol/l	35 (1.9)	23 (1.9)	12 (1.9)	
Missing	16 (0.9)	10 (0.8)	12 (0.9)	
BMI, mean (SD)	25.4 (5.7)	25.2 (5.4)	25.8 (6.5)	0.021
BMI, N (%)				0.010
< 25	843 (46)	568 (47)	275 (44)	
25–29.9	642 (35)	428 (36)	214 (34)	
≥ 30	342 (19)	200 (17)	142 (23)	
Missing	4 (0.2)	1 (0.1)	3 (0.5)	
Waist-hip ratio, mean (SD)	0.85 (0.09)	0.85 (0.09)	0.85 (0.09)	0.61
Waist-hip ratio – risk level, N (%)				0.002
1 (Green)	696 (38)	484 (41)	212 (34)	
2	541 (30)	360 (30)	181 (29)	
3	324 (18)	194 (16)	130 (21)	
4 (Red)	249 (14)	147 (12)	102 (16)	
Missing	21 (1)	12 (1)	9 (1)	
Physical activity – risk level, N (%)				0.042
1 (Green)	579 (32)	401 (34)	178 (28)	
2	488 (27)	312 (26)	176 (28)	
3	256 (14)	172 (14)	84 (13)	
4 (Red)	508 (28)	312 (26)	196 (31)	
Diet – risk level, N (%)				0.09
1 (Green)	687 (38)	463 (39)	224 (35)	
2	523 (29)	346 (29)	177 (28)	
3 (Red)	576 (31)	355 (30)	221 (35)	
Missing	45 (2)	33 (3)	12 (2)	
Alcohol – risk level, N (%)				0.001
1 (Green)	1481 (81)	977 (82)	504 (80)	
2	43 (2.3)	24 (2.0)	19 (3.0)	
3	174 (9.5)	122 (10)	52 (8.2)	
4 (Red)	70 (3.8)	32 (2.7)	38 (6.0)	
Missing	63 (3.4)	42 (3.5)	21 (3.3)	
Tobacco use – risk level, N (%)				<0.001
1 (Green)	1311 (72)	886 (74)	425 (67)	
2	286 (16)	184 (15)	102 (16)	
3	129 (7.0)	74 (6.2)	55 (8.7)	
4 (Red)	104 (5.7)	52 (4.3)	52 (8.2)	
Missing	1 (<0.1)	1 (<0.1)	0	
Life situation/psychosocial strain – risk level, N (%)				<0.001
1 (Green)	1716 (94)	1144 (96)	572 (90)	
2	107 (5.8)	50 (4.2 %)	57 (9.0)	
3 (Red)	7 (0.4)	2 (0.2)	5 (0.8)	
Missing	1 (<0.1)	1 (<0.1)	0	
Heredity, diabetes – risk level, N (%)				0.06
1 (Green)	1250 (68)	836 (70)	414 (65)	
2	444 (24)	283 (24)	161 (25)	
3 (Red)	87 (4.8)	48 (4.0)	39 (6.2)	
Missing	50 (2.7)	30 (2.5)	20 (3.2)	
Heredity, CVD – risk level, N (%)				0.20
1 (Green)	1490 (81)	986 (82)	504 (79)	
2	171 (9.3)	111 (9.3)	60 (9.5)	
3	97 (5.3)	58 (4.8)	39 (6.2)	
4 (Red)	55 (3.0)	30 (2.5)	25 (3.9)	
Missing	18 (1.0)	12 (1.0)	6 (0.9)	

Group comparison analyses showed significant differences between the group with high level of self-reported psychiatric symptoms compared with the rest of the study population, with a higher proportion of individuals who had the highest level of waist-hip ratio, physical inactivity, alcohol intake, tobacco use and psychosocial strain in the group with level 3 for psychiatric symptoms. There were no differences regarding heredity for diabetes or cardiovascular disease, blood pressure, fasting plasma glucose or serum cholesterol between the groups (Table 1).

A total of 518 (28 %) participants had previously registered psychiatric diagnoses (F-diagnoses according to the International Classification of Diseases ICD-10) during the last two years. Of these, 14 individuals had F20 (schizophrenia, schizotypal and delusional disorders) or F31 (bipolar disorder) registered at least once during their lifetime. More individuals with psychiatric diagnoses reported higher levels of psychiatric symptoms compared with individuals without registered psychiatric diagnoses (Table 2).

**Table 2:** Comparison in level of psychiatric symptoms on the Health Profile in individuals with or without psychiatric diagnoses.

Psychiatric diagnosis (Yes/No)	Level of psychiatric illness on the Health Profile		p-value*
	1–2 (low risk)	3 (high risk)	
No, N (%)	985 (53)	328 (18)	<0.001
Yes, N (%)	212 (12)	306 (17)	

*chi^2^ test.

We performed a subgroup analysis with a comparison between individuals, with or without psychiatric diagnosis, and the difference in cardiovascular risk factors between participants with psychiatric symptoms or not according to the Health Profile. The comparison showed that in the group without psychiatric diagnoses but who had self-reported psychiatric symptoms, there were significantly more participants with high levels of physical inactivity, tobacco use, alcohol, BMI, waist-hip ratio, and life situation (psychosocial strain) compared with the group reporting no psychiatric symptoms (level 1–2) (Supplementary Table 1). No such differences in lifestyle habits were found in individuals with previously diagnosed psychiatric diseases and different levels of self-reported psychiatric symptoms on the Health Profile.

The Health Dialogue generated more referrals or visits to other health care providers in the group with the highest level of psychiatric symptoms (32 %) compared to the rest of the study population (23 %), with significant differences (p < 0.05) for referrals regarding support for increased physical activity, alcohol counselling, sleep apnea syndrome, sleeping disorders, chest pain and psychiatric symptoms. There was no difference between the groups regarding detection or suspicion of hypertension, dyslipidemia, diabetes, or impaired serum glucose.

A logistic regression analysis was performed to study the association between different cardiovascular risk factors and the level of self-reported psychiatric symptoms. The analysis showed that physical inactivity (OR = 1.42), unhealthy diet (OR = 1.29), risky alcohol intake (OR = 2.3), tobacco use (OR = 2.8), life situation/psychosocial strain (OR = 5), higher BMI (OR = 1.9) and higher waist-hip ratio (OR = 1.58) were statistically significantly associated with high levels of psychiatric symptoms on the Health Profile (Table 3). The increased ORs remained after adjustment for sex, marital status, employment, and educational level. No associations were seen between high-risk levels for blood pressure, fasting glucose or cholesterol and the level for psychiatric symptoms, respectively.

**Table 3:** Regression analysis for the association between risk levels on the Health Profile and psychiatric symptoms. OR for psychiatric symptoms (level 3 on the Health Profile) are shown with 95% confidence interval.

Risk level on the Health Profile	Model 1 (unadjusted)		Model 2*		Model 3**	
	OR (95 % CI)		OR (95 % CI)		OR (95 % CI)	
Physical activity	Ref: 1 (Green)		Ref: 1 (Green)		Ref: 1 (Green)	
2	1.27	0.98; 1.64	1.24	0.96; 1.60	1.28	0.99; 1.67
3	1.10	0.80; 1.50	1.08	0.78; 1.48	1.15	0.83; 1.58
4 (Red)	1.42	1.10; 1.82	1.45	1.13; 1.87	1.48	1.15; 1.92
Dietary habits	Ref: 1 (Green)					
2	1.06	0.83; 1.35	1.04	0.81; 1.32	1.04	0.81; 1.33
3 (Red)	1.29	1.02; 1.62	1.30	1.03; 1.64	1.29	1.02; 1.64
Alcohol intake	Ref: 1 (Green)					
2	1.53	0.82; 2.82	1.52	0.81; 2.80	1.52	0.81; 2.83
3	0.83	0.58; 1.16	0.91	0.64; 1.28	0.84	0.59; 1.19
4 (Red)	2.30	1.42; 3.75	2.85	1.74; 4.69	2.22	1.35; 3.66
Tobacco use	Ref: 1 (Green)					
2	1.16	0.88; 1.51	1.47	1.10; 1.95	1.49	1.11; 1.98
3	1.55	1.07; 2.23	1.69	1.16; 2.45	1.63	1.11; 2.39
4 (Red)	2.08	1.39; 3.12	2.22	1.48; 3.34	2.18	1.43; 3.32
Life situation/ Psychosocial strain	Ref: 1 (Green)					
2	2.28	1.54; 3.39	2.31	1.56; 3.45		
3 (Red)	5.00	1.07; 35.0	5.48	1.16; 38.7		
BMI	Ref: < 25					
25–29.9	1.04	0.84; 1.29	1.16	0.93; 1.45	1.16	0.93; 1.46
≥ 30	1.47	1.14; 1.91	1.55	1.19; 2.01	1.52	1.16; 1.99
Waist-hip ratio	Ref: 1 (Green)					
2	1.15	0.90; 1.46	1.12	0.88; 1.43	1.15	0.90; 1.47
3	1.53	1.16; 2.01	1.42	1.08; 1.88	1.51	1.14; 2.00
4 (Red)	1.58	1.17; 2.14	1.51	1.11; 2.04	1.58	1.16; 2.14
Blood pressure	Ref: 1 (Green)					
2	0.95	0.74; 1.23	1.08	0.83; 1.40	1.10	0.84; 1.43
3	1.02	0.35; 2.70	1.20	0.41; 3.20	1.20	0.40; 3.22
4 (Red)	0.62	0.09; 2.72	0.70	0.10; 3.07	0.71	0.10; 3.18
Cholesterol	Ref: 1 (Green)					
2	1.00	0.81; 1.22	1.08	0.88; 1.33	1.07	0.87; 1.32
3	1.45	0.84; 2.46	1.87	1.08; 3.22	1.92	1.10; 3.33
4 (Red)	1.91	0.08; 48.3	2.06	0.08; 53.2	2.37	0.09; 61.1
F-plasma-glucose	Ref: ≤ 6 mmol/l					
6.1–6.9 mmol/l	0.68	0.45; 1.00	0.75	0.49; 1.10	0.76	0.50; 1.13
≥ 7 mmol/l	0.96	0.46; 1.91	1.14	0.54; 2.28	1.04	0.49; 2.13

*Model 2 is adjusted for sex. **Model 3 is adjusted for sex, marital status, employment, and educational level.

## 4 Discussion

### 4.1 Main results

Participants with the highest level of self-reported psychiatric symptoms had higher risk levels of waist-hip ratio, physical inactivity, alcohol intake, tobacco use, and life situation/psychosocial strain, compared to the rest of the study population. A sub-group analysis found the same significant differences in the group with high level of psychiatric symptoms on the Health Profile but without previously registered psychiatric diagnoses during the last two years. There was an association between an elevated level on the Health Profile for several lifestyle habits and high level of self-reported psychiatric symptoms, and the association remained significant after adjustments for sex, marital status, employment, and educational level. Our findings suggest that using a targeted health dialogue model could provide primary care settings with new opportunities to address and intervene on risk factors for cardiovascular disease in patients with self-reported psychiatric symptoms, as they seem to be a group with more unhealthy lifestyle habits compared with the rest of the study population. In a primary care under strain, individually tailored and targeted lifestyle interventions might be more cost-effective in the long run, having the potential to prevent or ameliorate both psychiatric and cardiovascular diseases. Besides the potential benefits on psychiatric health, modification of unhealthy behaviors might also reduce the risk of developing obesity, high blood pressure, diabetes, and other risk factors for cardiovascular disease. Regardless of the potential selection bias using the recruitment method in this study, our results indicate that individuals reporting psychiatric symptoms may be suitable for a targeted health dialogue in primary care.

### 4.2 Comparison with other studies

There is strong evidence that patients with both severe and common psychiatric problems have a higher risk of cardiometabolic disease compared to the rest of the population (1, 3, 4, 8–10, 13, 30). The results are consistent with findings in a Swedish study where patients with diagnosed psychiatric illness (depression, anxiety, sleeping disorder or stress) were included opportunistically after contact with primary care and screened for unhealthy lifestyle habits with a targeted Health Dialogue (Pikkemaat et al., 2022).

Our results show that even individuals with self-reported psychiatric symptoms detected with lifestyle screening and no previous history of psychiatric illness have higher levels of cardiovascular risk factors including both unhealthy lifestyle habits and anthropometric measurements such as higher BMI and waist-hip ratio. To our knowledge, there are no previous studies focusing on the association between cardiovascular risk factors and self-reported psychiatric symptoms detected by screening (not seeking primary care for the symptomatology). The increase of the cardiometabolic risk in people with psychiatric illness is often attributed to medication side effects such as weight gain, including antipsychotics or antidepressants (Firth et al., 2019). Our results indicate that the risk is increased even in relatively young individuals without previously diagnosed psychiatric disease, but who are assessed to have higher levels of self-reported psychiatric symptoms with a structured screening instrument. There were no differences between the groups, with or without mental illness, regarding blood pressure, cholesterol, or fasting plasma glucose. The findings suggest that early detection of high-risk levels in different lifestyle habits, and interventions with a focus on modifying unhealthy behaviors could prevent the development of cardiometabolic changes and later CVD or diabetes in individuals with self-reported psychiatric symptoms.

### 4.3 Strengths and weaknesses

The study population was recruited from a large geographic area with a population with high demographic diversity (including socioeconomic characteristics) of both men and women. The recruitment method offered a high generalizability of the results, which is a strength of the study. We found no other studies aiming to investigate the association between lifestyle habits and psychiatric symptoms in 40-year-old individuals, and this is also a strength of the study. A total of 21 % of the invited individuals agreed to participate in the research study. There are risks for selection bias as healthier individuals, who are more interested in a healthy lifestyle, might have answered the invitation to a targeted Health Dialogue. This is a major limitation of this study. Previous research indicates that patients suffering from mental illness lack interest in preventive care, meaning that they are less prone to respond to screening invitations (Stumbo et al., 2018). There is therefore a possibility that the study population is not entirely representative of all 40-year-old individuals in the region, and the percentage of individuals with self-reported psychiatric symptoms is underestimated. As previously shown, non-participation in a lifestyle screening invitation might depend on a recent health examination at work, or high health care consumption in general (Persson et al., 1994), thus indicating that the individuals who declined participation might need more attention. The assessment of psychiatric symptoms on the Health Profile was based on rather general questions about anxiety, depression, sleeping problems or stress. The analysis showed, however, that individuals with low self-reported levels of psychiatric symptoms had fewer previously diagnosed psychiatric diagnoses, indicating that the Health Profile is useful when assessing the risk for psychiatric illness. Except for the blood tests and the anthropometric measurements, the assessment is based on self-reported habits, and this is a limitation of the study. For example, it has been shown that self-reported alcohol consumption is underestimated in national surveys compared to sales or taxation data (Stockwell et al., 2018). It is also common for people to underestimate their energy intake when data about dietary habits is self-reported (Grandjean, 2012). On the contrary, physical activity level is often overestimated in questionnaires compared with accelerometer data (Cerin et al., 2016).

Another limitation of the study is the cross-sectional study design. It is difficult to establish if unhealthy lifestyle habits cause/aggravate psychiatric illness, or if psychiatric diseases or symptoms lead to poor lifestyle choices. Our study found an association between unhealthy lifestyle and psychiatric symptoms. However, the causality is unclear.

## 5 Conclusion

Unhealthy lifestyle habits were associated with psychiatric symptoms in 40-year-old individuals assessed with targeted Health Dialogues in a primary care context. Organized screening might contribute to the early detection of modifiable risk factors for cardiovascular disease, and individuals with psychiatric symptoms should be prioritized for screening of unhealthy lifestyle behaviors.

## CRediT authorship contribution statement

**Veronica Milos Nymberg:** . **Peter Nymberg:** Writing – review & editing, Methodology, Conceptualization. **Miriam Pikkemaat:** Writing – review & editing, Conceptualization. **Susanna Calling:** Writing – review & editing. **Emelie Stenman:** Writing – review & editing, Investigation. **Anton Grundberg:** Writing – review & editing, Formal analysis, Data curation. **J. Gustav Smith:** Writing – review & editing. **Kristina Sundquist:** Writing – review & editing, Resources, Investigation, Funding acquisition.

## Declaration of competing interest

The authors declare that they have no known competing financial interests or personal relationships that could have appeared to influence the work reported in this paper.

## Data Availability

Data will be made available on request.
