# Supplementary material for: Lifestyle and cardiovascular risk factors in a Swedish primary care population with self-reported psychiatric symptoms
Source: Prev Med Rep. 2023 Dec 12;37:102547. doi: 10.1016/j.pmedr.2023.102547 (PMC10761795; doi:10.1016/j.pmedr.2023.102547)
Supplement: Supplementary data 1 [file mmc1.docx]

**Supplementary Table 1.** Group comparison analysis between participants with or without previous psychiatric diagnoses and the cardiovascular risk, separated by self-reported level for psychiatric symptoms on the Health Profile.

|  | No previous F-diagnosis  n = 1313 | | | Previous F-diagnosis  n = 518 | | |
| --- | --- | --- | --- | --- | --- | --- |
|  | Level psychiatric illness | |  | Level psychiatric illness | |  |
| Variable | 1-2 | 3 | p-value | 1-2 | 3 | p-value |
| Sex |  |  | <0.001 |  |  | 0.08 |
| Men, N (%) | 500 (51) | 127 (39) |  | 89 (42) | 105 (34) |  |
| Women, N (%) | 485 (49) | 201 (61) |  | 123 (58) | 201 (66) |  |
| Blood pressure– risk level, N (%) |  |  | 0.98 |  |  | 0.39 |
| 1 (Green) | 800 (81) | 269 (82) |  | 170 (80) | 249 (81) |  |
| 2 | 166 (17) | 54 (17) |  | 42 (20) | 52 (17) |  |
| 3 | 11 (1.1) | 3 (0.9) |  | 0 (0) | 3 (1.0) |  |
| 4 (Red) | 6 (0.6) | 1 (0.3) |  | 0 (0) | 1 (0.3) |  |
| Missing | 2 (0.2) | 1 (0.3) |  | 0 | 1 (0.3) |  |
| Cholesterol– risk level, N (%) |  |  | 0.95 |  |  | 0.17 |
| 1 (Green) | 601 (61) | 201 (62) |  | 126 (59) | 180 (59) |  |
| 2 | 348 (36) | 114 (35) |  | 80 (38) | 110 (36) |  |
| 3 | 30 (3.1) | 11 (3.4) |  | 3 (1.4) | 14 (4.6) |  |
| 4 (Red) | 1 (0.1) | 0 |  | 0 (0% | 1 (0.3) |  |
| Missing | 5 (0.5) | 2 (0.6) |  | 3 (1.4) | 1 (0.3) |  |
| F-plasma-glucose, N (%) |  |  | 0.29 |  |  | 0.80 |
| ≤ 6 mmol/l | 874 (89) | 300 (91) |  | 193 (91) | 280 (92) |  |
| 6.1 – 6.9 mmol/l | 83 (8.) | 19 (5.8) |  | 14 (6.6) | 17 (5.6) |  |
| ≥ 7 mmol/l | 20 (2.0) | 6 (1.8) |  | 3 (1.4) | 6 (2.0) |  |
| Missing | 8 (0.8) | 3 (0.9) |  | 2 (0.9) | 3 (1.0) |  |
| BMI, N (%) |  |  | 0.033 |  |  | 0.71 |
| < 25 | 478 (49) | 142 (43) |  | 90 (42) | 132 (43) |  |
| 25 – 29.9 | 350 (36) | 113 (34) |  | 78 (37) | 102 (33) |  |
| ≥ 30 | 156 (16) | 72 (22) |  | 44 (21) | 70 (23) |  |
| Missing | 1 (0.1) | 1 (0.3) |  | 0 | 2 (0.7) |  |
| Waist-hip-ratio– risk level, N (%) |  |  | 0.023 |  |  | 0.96 |
| 1 (Green) | 415 (42) | 118 (36) |  | 69 (33) | 94 (31) |  |
| 2 | 299 (30) | 89 (27) |  | 61 (29) | 92 (30) |  |
| 3 | 150 (15) | 63 (19) |  | 44 (21) | 67 (22) |  |
| 4 (Red) | 112 (11) | 52 (16) |  | 35 (17) | 50 (16) |  |
| Missing | 9 (0.9) | 6 (1.8) |  | 3 (1.4) | 3 (1.0) |  |
| Physical activity– risk level, N (%) |  |  | 0.045 |  |  | 0.73 |
| 1 (Green) | 337 (34) | 91 (28) |  | 64 (30) | 87 (28) |  |
| 2 | 256 (26) | 82 (25) |  | 56 (26) | 94 (31) |  |
| 3 | 150 (15) | 51 (16) |  | 22 (10) | 33 (11) |  |
| 4 (Red) | 242 (25) | 104 (32) |  | 70 (33) | 92 (30) |  |
| Dietary habits– risk level, N (%) |  |  | 0.38 |  |  | 0.64 |
| 1 (Green) | 387 (39) | 122 (37) |  | 76 (36) | 102 (33) |  |
| 2 | 288 (29) | 90 (27) |  | 58 (27) | 87 (28) |  |
| 3 (Red) | 287 (29) | 109 (33) |  | 68 (32) | 112 (37) |  |
| Missing | 23 (2.3) | 7 (2.1) |  | 10 (4.7) | 5 (1.6) |  |
| Alcohol intake– risk level, N (%) |  |  | 0.011 |  |  | 0.11 |
| 1 (Green) | 799 (81) | 272 (83) |  | 178 (84) | 232 (76) |  |
| 2 | 20 (2.0) | 9 (2.7) |  | 4 (1.9) | 10 (3.3) |  |
| 3 | 109 (11) | 22 (6.7) |  | 13 (6.1) | 30 (9.8) |  |
| 4 (Red) | 24 (2.4) | 17 (5.2) |  | 8 (3.8) | 21 (6.9) |  |
| Missing | 33 (3.4) | 8 (2.4) |  | 9 (4.2) | 13 (4.2) |  |
| Tobacco use– risk level, N (%) |  |  | 0.030 |  |  | 0.66 |
| 1 (Green) | 754 (77) | 244 (74) |  | 132 (62) | 181 (59) |  |
| 2 | 149 (15) | 42 (13) |  | 35 (17) | 60 (20) |  |
| 3 | 56 (5.7) | 23 (7.0) |  | 18 (8.5) | 32 (10) |  |
| 4 (Red) | 26 (2.6) | 19 (5.8) |  | 26 (12) | 33 (11) |  |
| Missing | 0 | 0 |  | 1 (0.5) | 0 |  |
| Life situation/ Psychosocial strain– risk level, N (%) |  |  | 0.004 |  |  | 0.29 |
| 1 (Green) | 952 (97) | 306 (93) |  | 192 (91) | 266 (87) |  |
| 2 | 32 (3.2) | 18 (5.5) |  | 18 (8.5) | 39 (13) |  |
| 3 (Red) | 1 (0.1) | 4 (1.2) |  | 1 (0.5) | 1 (0.3) |  |
| Missing | 0 | 0 |  | 1 (0.5) | 0 |  |
| Heredity, diabetes – risk level, N (%) |  |  | 0.042 |  |  | 0.93 |
| 1 (Green) | 692 (70) | 211 (64) |  | 144 (68) | 203 (66) |  |
| 2 | 235 (24) | 88 (27) |  | 48 (23) | 73 (24) |  |
| 3 (Red) | 34 (3.5) | 20 (6.1) |  | 14 (6.6) | 19 (6.2) |  |
| Missing | 24 (2.4) | 9 (2.7) |  | 6 (2.8) | 11 (3.6) |  |
| Heredity, CVD – risk level, N (%) |  |  | 0.41 |  |  | 0.13 |
| 1 (Green) | 810 (82) | 275 (84) |  | 176 (83) | 229 (75) |  |
| 2 | 95 (9.6) | 26 (7.9) |  | 16 (7.5) | 34 (11) |  |
| 3 | 47 (4.8) | 12 (3.7) |  | 11 (5.2) | 27 (8.8) |  |
| 4 (Red) | 24 (2.4) | 12 (3.7) |  | 6 (2.8) | 13 (4.2) |  |
| Missing | 9 (0.9) | 3 (0.9) |  | 3 (1.4) | 3 (1.0) |  |
